# Bioinspired polysaccharide-based nanocomposite membranes with robust wet mechanical properties for guided bone regeneration

**DOI:** 10.1093/nsr/nwad333

**Published:** 2024-01-02

**Authors:** Jian-Hong Xiao, Zhen-Bang Zhang, JiaHao Li, Si-Ming Chen, Huai-Ling Gao, YinXiu Liao, Lu Chen, ZiShuo Wang, YiFan Lu, YuanZhen Hou, HengAn Wu, DuoHong Zou, Shu-Hong Yu

**Affiliations:** Department of Chemistry, New Cornerstone Science Laboratory, Institute of Biomimetic Materials & Chemistry, Anhui Engineering Laboratory of Biomimetic Materials, Division of Nanomaterials & Chemistry, Hefei National Research Center for Physical Sciences at the Microscale, University of Science and Technology of China, Hefei 230026, China; Department of Dental Implant Center, Stomatologic Hospital and College, Key Laboratory of Oral Diseases Research of Anhui Province, Anhui Medical University, Hefei 230032, China; Department of Chemistry, New Cornerstone Science Laboratory, Institute of Biomimetic Materials & Chemistry, Anhui Engineering Laboratory of Biomimetic Materials, Division of Nanomaterials & Chemistry, Hefei National Research Center for Physical Sciences at the Microscale, University of Science and Technology of China, Hefei 230026, China; CAS Key Laboratory of Mechanical Behavior and Design of Materials, Department of Modern Mechanics, CAS Center for Excellence in Complex System Mechanics, University of Science and Technology of China, Hefei 230027, China; Department of Chemistry, New Cornerstone Science Laboratory, Institute of Biomimetic Materials & Chemistry, Anhui Engineering Laboratory of Biomimetic Materials, Division of Nanomaterials & Chemistry, Hefei National Research Center for Physical Sciences at the Microscale, University of Science and Technology of China, Hefei 230026, China; Department of Chemistry, New Cornerstone Science Laboratory, Institute of Biomimetic Materials & Chemistry, Anhui Engineering Laboratory of Biomimetic Materials, Division of Nanomaterials & Chemistry, Hefei National Research Center for Physical Sciences at the Microscale, University of Science and Technology of China, Hefei 230026, China; CAS Key Laboratory of Mechanical Behavior and Design of Materials, Department of Modern Mechanics, CAS Center for Excellence in Complex System Mechanics, University of Science and Technology of China, Hefei 230027, China; Department of Oral Surgery, College of Stomatology, National Clinical Research Center for Oral Diseases, Shanghai Key Laboratory of Stomatology, Shanghai Research Institute of Stomatology, Shanghai Ninth People's Hospital Affiliated to Shanghai Jiao Tong University School of Medicine, Shanghai 200001, China; Department of Oral Surgery, College of Stomatology, National Clinical Research Center for Oral Diseases, Shanghai Key Laboratory of Stomatology, Shanghai Research Institute of Stomatology, Shanghai Ninth People's Hospital Affiliated to Shanghai Jiao Tong University School of Medicine, Shanghai 200001, China; Department of Oral Surgery, College of Stomatology, National Clinical Research Center for Oral Diseases, Shanghai Key Laboratory of Stomatology, Shanghai Research Institute of Stomatology, Shanghai Ninth People's Hospital Affiliated to Shanghai Jiao Tong University School of Medicine, Shanghai 200001, China; Department of Dental Implant Center, Stomatologic Hospital and College, Key Laboratory of Oral Diseases Research of Anhui Province, Anhui Medical University, Hefei 230032, China; CAS Key Laboratory of Mechanical Behavior and Design of Materials, Department of Modern Mechanics, CAS Center for Excellence in Complex System Mechanics, University of Science and Technology of China, Hefei 230027, China; CAS Key Laboratory of Mechanical Behavior and Design of Materials, Department of Modern Mechanics, CAS Center for Excellence in Complex System Mechanics, University of Science and Technology of China, Hefei 230027, China; Department of Dental Implant Center, Stomatologic Hospital and College, Key Laboratory of Oral Diseases Research of Anhui Province, Anhui Medical University, Hefei 230032, China; Department of Oral Surgery, College of Stomatology, National Clinical Research Center for Oral Diseases, Shanghai Key Laboratory of Stomatology, Shanghai Research Institute of Stomatology, Shanghai Ninth People's Hospital Affiliated to Shanghai Jiao Tong University School of Medicine, Shanghai 200001, China; Department of Chemistry, New Cornerstone Science Laboratory, Institute of Biomimetic Materials & Chemistry, Anhui Engineering Laboratory of Biomimetic Materials, Division of Nanomaterials & Chemistry, Hefei National Research Center for Physical Sciences at the Microscale, University of Science and Technology of China, Hefei 230026, China; Institute of Innovative Materials (I2M), Department of Chemistry, Department of Materials Science and Engineering, Southern University of Science and Technology, Shenzhen 518055, China

**Keywords:** bioinspiration, heterogeneous crosslink-and-hydration, dual-scale network, nanocomposite membrane, wet mechanical properties, guided bone regeneration

## Abstract

Polysaccharide-based membranes with excellent mechanical properties are highly desired. However, severe mechanical deterioration under wet conditions limits their biomedical applications. Here, inspired by the structural heterogeneity of strong yet hydrated biological materials, we propose a strategy based on heterogeneous crosslink-and-hydration (HCH) of a molecule/nano dual-scale network to fabricate polysaccharide-based nanocomposites with robust wet mechanical properties. The heterogeneity lies in that the crosslink-and-hydration occurs in the molecule-network while the stress-bearing nanofiber-network remains unaffected. As one demonstration, a membrane assembled by bacterial cellulose nanofiber-network and Ca^2+^-crosslinked and hydrated sodium alginate molecule-network is designed. Studies show that the crosslinked-and-hydrated molecule-network restricts water invasion and boosts stress transfer of the nanofiber-network by serving as interfibrous bridge. Overall, the molecule-network makes the membrane hydrated and flexible; the nanofiber-network as stress-bearing component provides strength and toughness. The HCH dual-scale network featuring a cooperative effect stimulates the design of advanced biomaterials applied under wet conditions such as guided bone regeneration membranes.

## Teaser

Polysaccharide-based nanocomposite membranes with excellent mechanical properties can be produced via one bioinspired approach, indicating tremendous development potential for biomedical applications such as guided bone regeneration.

## INTRODUCTION

With increasing demand for the repair of tissue defects in the clinic, various biomaterials have been developed to provide specific biological functions [1–6]. Among them, membrane materials are particularly important for *in vivo* treatment, e.g. hydrogel membranes for the repair of damaged tendons [7,8], antiadhesion membranes for the prevention of postoperative tissue adhesion [7,9,10], as well as barrier membranes for guided bone regeneration (GBR) [11–13]. Currently, clinically used biomedical membranes are mainly derived from animal acellular tissues (such as small intestinal submucosa and porcine dermis) [14–16] and collagen products like Bio-Gide membrane (the current gold standard in the clinic for GBR) [17]. However, animal-derived membranes may cause risks of immune rejection *in vivo* due to incomplete decellularization [16,18]. In recent years, many polysaccharides (such as cellulose, sodium alginate (SA), chitosan (CS), and chitin) have attracted increasing attention in the fabrication of new-style biomedical membranes due to their excellent biocompatibility, non-toxicity, bioactivity, and biodegradability [19–22]. For instance, SA and CS, two common natural polysaccharides derived from algae and crustacean shells, respectively, have been extensively utilized for tissue engineering and wound dressings, yet their mechanical properties are less satisfactory [21,23–30].

To expand the applications of these polysaccharide-based membranes, nanofillers [13,31] and chemical crosslinking [25,32,33] are often used to improve their mechanical properties. Though membranes with high mechanical strength in a dry state have been successfully fabricated, their structures are unstable under wet conditions. This is mainly attributed to the high hydrophilicity of the fundamental polysaccharides [34]. Upon hydration, H_2_O molecules penetrate polymer matrices and compete for hydrogen bonds between polymer chains, leading to nonnegligible swelling and mechanical deterioration of materials [35,36]. Furthermore, *in vivo* applications are often accompanied by complex stress conditions, such as stretching, suturing, and tearing [37]. Therefore, in addition to possessing excellent biological functions, ideal biomedical membranes should also have sufficient strength and damage tolerance to resist complex stress under wet physiological conditions. However, achieving the combination of robust wet mechanical properties and biological functions within polysaccharide-based membranes remains challenging. More specifically, how to improve the wet mechanical properties of polysaccharide-based membranes is particularly critical and is worth exploring.

Nature often provides solutions to problems in reality. Hydrated biological materials such as skin and fish scales, although composed of meager components, always exhibit superior mechanical properties and stability under wet physiological conditions due to their hierarchical and heterogeneous fiber-based structures [38,39]. For example, tiny elastic fibers and large collagen fibers that make up the dermis of skin are spatially intertwined and suspended in gel-like wet substances, the special composite structure and microenvironment allow elastic fibers to endow skin with good flexibility and collagen fibers to endow skin with high tensile strength, respectively [38]. The synergetic effect of elastic fibers and collagen fibers is remarkable. Overall, the site-specific attributes (such as composition, structural motif, and microenvironment) and the resulting hierarchical and heterogeneous structures accompanied by excellent mechanical properties of biological materials provide abundant inspiration for the design of high-performance materials [40–42].

**Figure 1.**
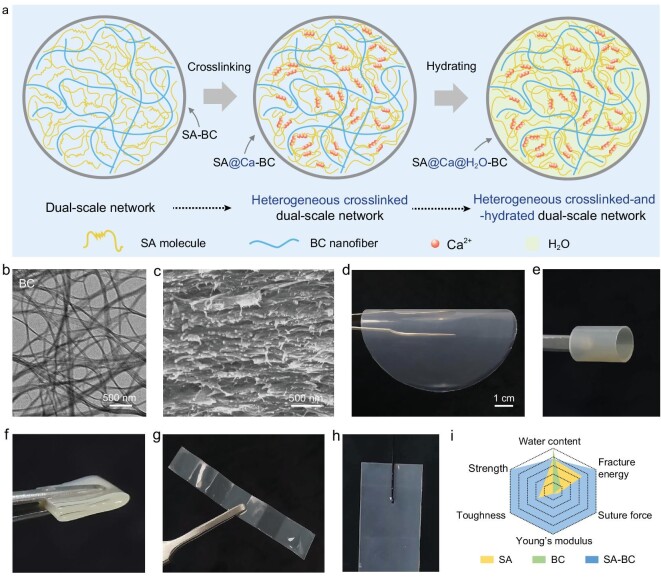
Structure, material and properties of heterogeneous crosslinked-and-hydrated (HCH) dual-scale network. (a) Schematic illustration of HCH dual-scale network designed by sequential procedures. (b) TEM image of BC nanofiber, showing a typical fibrous network structure. (c) SEM image of the cross-section of HCH dual-scale network SA@Ca@H_2_O-BC membrane (SA : BC = 10 : 4), implying the dual-scale nanofiber-network and molecule-network. (d–g) Photographs of SA@Ca@H_2_O-BC membrane processed into different shapes, presenting the membrane's flexibility. (h) Photograph of SA@Ca@H_2_O-BC membrane penetrated by needle and thread. No secondary crack exists near the needle-induced hole, demonstrating the membrane's robustness. (i) Properties comparison of SA@Ca@H_2_O-BC membrane, SA@Ca@H_2_O membrane and BC@Ca@H_2_O membrane, showing the superiority of HCH dual-scale network. ‘@Ca@H_2_O’ is omitted for brevity.

Herein, we propose a bioinspired mechanical reinforcement strategy based on heterogeneous crosslink-and-hydration (HCH) of a molecule/nano dual-scale network to fabricate polysaccharide-based nanocomposite membranes with robust wet mechanical properties (Fig. 1a). The heterogeneity lies in that crosslink-and-hydration selectively occurs in the small molecule-scale network. Under such circumstances, the undisturbed nanoscale fiber network can bear stresses for providing mechanical robustness while the molecule-scale network makes the membrane flexible. As one demonstration, a membrane comprising bacterial cellulose (BC) nanofiber-network and Ca^2+^-crosslinked and hydrated SA (expressed as SA@Ca@H_2_O) molecule-network is fabricated (Fig. 1b and c). Systematic characterizations reveal that the membrane (expressed as SA@Ca@H_2_O-BC membrane) exhibits shape flexibility (Fig. 1d–h) and excellent wet mechanical properties compared with SA@Ca@H_2_O membrane (with molecule-network) and BC@Ca@H_2_O membrane (with nanofiber-network) (Fig. 1i), reflecting the advantages of an HCH dual-scale network. With suitable functionalization, the HCH dual-scale network SA@Ca@H_2_O-BC membrane exhibits biomedical application potential (such as GBR), demonstrated by *in vitro* cell tests and *in vivo* dog alveolar bone repair experiments.

## RESULTS AND DISCUSSION

### Fabrication and characterization of HCH dual-scale network SA@Ca@H_2_O-BC membrane

SA molecules and BC nanofibers are spatially mixed in the liquid environment to preliminarily construct an interweaved composite structure (Fig. 1a). After solvent evaporation, a SA-BC binary membrane can be obtained. By using a scanning electron microscope (SEM), SA and BC are found to be interpenetration-assembled in the membrane (Fig. 1c), confirming the dual-scale network design (including interweaving-coexisted obvious nanofiber-networks and comprehensible molecule-networks). Here, the mechanically optimized dual-scale network nanocomposite membrane (Fig. S1 in the online supplementary file) along with the single-scale network SA membrane and BC membrane is used to explore mechanical behaviors and mechanisms under wet conditions. We put these membranes into CaCl_2_ solution to stabilize corresponding networks (via Ca-COO and Ca-OH interaction) then dry them and put them into H_2_O for hydration. From the Fourier Transform Infrared (FTIR) spectra (Fig. S2), we can see that after introducing Ca^2+^, the characteristic peaks of COO and OH are shifted, which indicates that Ca^2+^ can affect COO and OH. The sequential steps of crosslinking and hydrating relate to the heterogeneous regulation that we are concerned with.

All membranes can maintain structural integrity. The H_2_O-absorption ratio of SA@Ca@H_2_O-BC@Ca@H_2_O membrane, SA@Ca@H_2_O membrane, and BC@Ca@H_2_O membrane are 140.2%, 124.4%, and 443.4%, respectively (Fig. S3). The difference in H_2_O-absorption capacity partly indicates the stronger coordination ability of Ca-COO than Ca-OH, and further indicates that Ca^2+^ mainly distributes in the SA molecule-network to form an SA@Ca molecule-network. The preformed SA@Ca molecule-network confined among the BC nanofiber-network can compact the global system and restrict the BC nanofiber-network from excessively absorbing H_2_O. Thus, SA@Ca@H_2_O-BC@Ca@H_2_O can be simply expressed as SA@Ca@H_2_O-BC.

**Figure 2.**
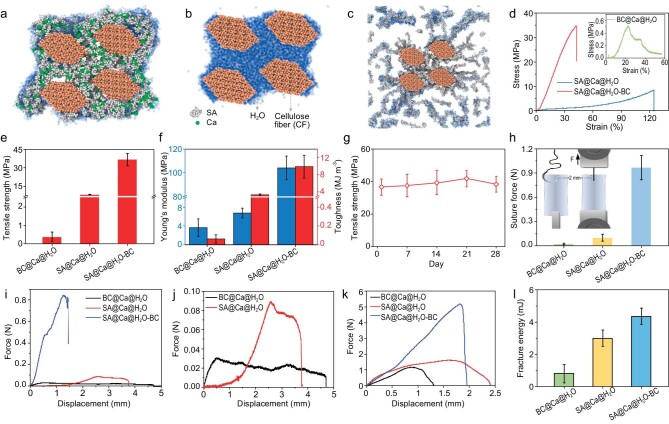
H_2_O molecule penetration simulations and mechanical superiority of SA@Ca@H_2_O-BC membrane. (a–c) Simulated diagrams of SA@Ca-CFs (a), pure CFs (b) and SA-CFs (c) at 4 ns. The green, gray, brown and blue spheres represent Ca, SA, CF and H_2_O, respectively. (d–f) Typical stress-strain curves, tensile strength, Young's modulus and toughness of SA@Ca@H_2_O-BC membrane, SA@Ca@H_2_O membrane, and BC@Ca@H_2_O membrane, showing the superiority of SA@Ca@H_2_O-BC membrane. (g) Tensile strength stability of SA@Ca@H_2_O-BC membrane, showing that the tensile strength of the membrane is relatively stable over a period of time. (h) Suture force of membranes. The inset shows the diagram details of the suture experiment (such as loading direction and sample size to be scratched). (i and j) Typical suture force-displacement curves of membranes. The suture force of SA@Ca@H_2_O membrane and BC@Ca@H_2_O membrane are too low, so we take them out separately to make Fig. 2j. (k) Typical tear force-displacement curves of membranes. (l) Tear fracture energy of membranes.

To study the role of Ca^2+^ and H_2_O molecule dynamic penetration, relevant models were constructed. A previous study shows that the bonding strength of bridging points (junctions) of the disordered network can determine the network's mechanical properties [43]. Therefore, models with four cellulose-fibers (CFs) indicating the nanofiber-network's bridging point are constructed (Fig. 2a–c). The interspace of the CFs is designed to be larger than the size of an H_2_O molecule and comparable with the size of CF, which is roughly consistent with experimental practice considering the size and interspace of BC nanofibers (Fig. 1b and c). Simulation reveals that when the stable SA@Ca is located in the interspace of CFs, outer H_2_O molecules cannot massively flood into the interspace (Fig. 2a and Fig. S4a). The result is different from that of pure CFs (H_2_O molecules fill the interspace of CFs) (Fig. 2b and Fig. S4b) and SA-CFs (H_2_O molecules dissolve SA and disintegrate composite structure) (Fig. 2c).

We further show the H_2_O penetration curve in Fig. S4c. It can be observed that pure CFs exhibit a steeper penetration trend before 1 ns and contain more H_2_O molecules in 4 ns (2.5 times as much as SA@Ca-CFs). This suggests that pure CFs exhibit a higher rate of H_2_O penetration, with H_2_O molecules more easily entering the interspace of CFs. Additionally, H_2_O distribution is investigated to better understand the final state of CFs@H_2_O and SA@Ca@H_2_O-CFs (Fig. S4d and e). With the radius of the circle increasing, the number of H_2_O molecules within CFs@H_2_O is significantly greater, which means that the distribution of H_2_O molecules is closer to the center and that the interspace of pure CFs is filled with bulk H_2_O (Fig. S4d and e). Principally, non-covalent interface plays a significant role in the mechanical properties of materials [44,45] and bulk H_2_O in the interspace of CFs can hardly transfer stress [46]. As a result, pure CFs soaked in H_2_O would exhibit poor mechanical properties compared with SA@Ca@H_2_O-CFs. The simulation results of three kinds of material systems display different H_2_O penetration situations and can support the experimental differences in H_2_O absorption. Overall, the formed SA@Ca@H_2_O molecule-network can avoid severe interaction between H_2_O and the BC nanofiber-network. In other words, the molecule-network serves as a barrier to massive H_2_O penetration and can transfer stress efficiently between interfaces at the bridging points of BC nanofiber-network.

### Mechanical properties of HCH dual-scale network SA@Ca@H_2_O-BC membrane

Uniaxial tensile test is performed to investigate the loading capacity of the HCH dual-scale network SA@Ca@H_2_O-BC membrane under wet conditions (Fig. 2d). In contrast to the dry mechanical results, the BC@Ca@H_2_O membrane here can hardly resist external load; compared with the SA@Ca@H_2_O membrane, the HCH dual-scale network SA@Ca@H_2_O-BC membrane exhibits excellent strength (36.72 MPa), Young's modulus (103.97 MPa) and toughness (9.89 MJ m^−3^) (Fig. 2e and f), although it has a relatively high H_2_O-absorption ratio (140.2% vs 124.4%) (Fig. S3). Moreover, the HCH dual-scale network SA@Ca@H_2_O-BC membrane exhibits mechanical stability during long-term (four weeks) immersion in H_2_O, indicating the equilibrium of crosslinking and hydrating within the HCH dual-scale network (Fig. 2g) which is consistent with that of the simulated dynamic penetration of H_2_O molecules (Fig. S4c). Not limited to uniaxial tensile tests, complex-loaded suture and tear tests also demonstrate the mechanical superiority of the HCH dual-scale network under wet conditions. As shown in Fig. 2h–j, the typical suturing load-displacement curve of the HCH dual-scale network SA@Ca@H_2_O-BC membrane is much higher than the others; the suture pullout force is 0.96 N, outperforming the SA@Ca@H_2_O membrane (0.1 N) and the BC@Ca@H_2_O membrane (0.02 N). In addition, the tearing fracture energy of the HCH dual-scale network SA@Ca@H_2_O-BC membrane is 4.36 mJ, which is higher than that of the BC@Ca@H_2_O membrane (3.0 mJ) and the SA@Ca@H_2_O membrane (0.81 mJ) (Fig. 2k and l).

**Figure 3.**
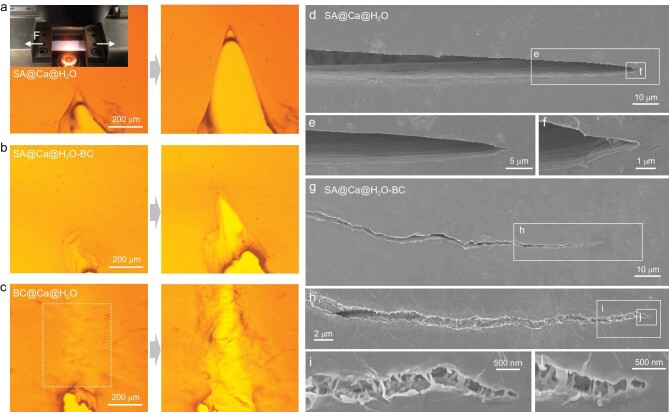
Crack propagation of membranes under *in-situ* loading, presenting different cracks on the pre-notched materials. (a–c) Photographs of SA@Ca@H_2_O membrane (a), SA@Ca@H_2_O-BC membrane (b), and BC@Ca@H_2_O (c) during *in-situ* loading under a microscope. Inset (a) is the *in-situ* loading equipment (loading direction is horizontal). (d–j) SEM images of SA@Ca@H_2_O membrane (d–f) and SA@Ca@H_2_O-BC membrane (g–j) under *in-situ* loading.

We specifically study the crack propagation that relates to toughening mechanisms. Under *in-situ* tensile loading, different cracks can be found on the pre-notched materials (Fig. 3, Movies S1–S3). SA@Ca@H_2_O membrane exhibits a smooth and straight crack with no bridge (Fig. 3a, d–f, Movie S1). On the contrary, the HCH dual-scale network SA@Ca@H_2_O-BC membrane exhibits large-scale crack deflection and extensive nanofiber bridge on the wake of the crack tip (Fig. 3b, g–j, Movie S2), which serves to dissipate crack tip stress and indicates extrinsic toughening [47,48]. Although the phenomenon of nanofiber bridging and interweaving is obvious (Fig. 3c), BC@Ca@H_2_O membrane is unable to resist crack propagation due to the severe penetration or lubrication by H_2_O molecules and, therefore, exhibits global catastrophic failure (white box in Fig. 3c indicates pre-failure, Movie S3) which corroborates the tested mechanical results (Fig. 2d–l). Overall, compared to the single-scale network (SA molecule-network or BC nanofiber-network), the HCH dual-scale network structure can play the role of mechanical reinforcement under wet conditions, endowing nanocomposites with excellent wet mechanical properties.

The heterogeneity-distributed Ca^2+^ is presumed to lay the mechanical foundation of the HCH dual-scale network nanocomposite. To further verify the role of Ca^2+^, we replace H_2_O (for hydration) with PBS solution (containing phosphates and monovalent metal ions) that can weaken Ca-COO coordination via capturing Ca^2+^ [49,50]. Studies show that the H_2_O-absorption ratio of the membrane in PBS (885.7%) is much higher than that in H_2_O (140.2%) (Fig. S5a). The higher swelling degree and lower mechanical properties of the membrane in PBS (Figs S5 and S6) indirectly confirm that Ca^2+^ helps restrict massive H_2_O penetration and that the resulting SA@Ca@H_2_O molecule-network with little H_2_O enables strong bridging points of the BC nanofiber-network. The simulation of H_2_O molecules penetrating SA-CFs also clearly reflects the role of Ca^2+^. Specifically, once Ca^2+^ is removed, H_2_O molecules can dissolve SA and disintegrate the composite structure (Fig. 2c), thus weakening mechanical properties.

### Universality of the bioinspired mechanical reinforcement strategy

The bioinspired mechanical reinforcement strategy can be extended from three aspects: molecule-matrix design, nano-reinforcement design, and the style of crosslink. For example, from the point of view of matrix design, SA can be replaced by pectin, sodium carboxymethyl cellulose, and chitosan; from the point of view of reinforcement design, BC can be replaced by lignocellulosic nanofibers, xonotlite nanofibers, and ultralong hydroxyapatite nanofibers; from the point of view of crosslink, Ca^2+^ can be replaced by genipin. It can be expected that many high-performance nanocomposite membranes can be realized. Here, to further experimentally verify the universality of the strategy mentioned above, using flexible xonotlite (CaSi) nanofibers (Fig. S7a) instead of BC nanofiber reinforcements, we can fabricate another high-performance nanocomposite membrane (SA@Ca@H_2_O-CaSi). As shown in Fig. S7b and c, the SA@Ca@H_2_O-CaSi nanocomposite membrane exhibits relatively excellent mechanical properties compared to the SA@Ca@H_2_O and the CaSi@Ca@H_2_O, highlighting the superiority of the HCH dual-scale network. Note that, the CaSi@Ca@H_2_O membrane is highly fragile (too weak), and its mechanical data cannot be shown.

### Design of HCH dual-scale network SA@Ca@H_2_O-BC membrane for GBR

The HCH dual-scale network-enabled superior mechanical properties under wet conditions make the SA-BC membrane a promising candidate for biomedical applications (from this subsection and beyond, we simplistically call SA@Ca@H_2_O-BC as SA-BC). As a primary demonstration of the GBR membrane, the SA-BC membrane elucidates three unique structure-induced advantages. First, its sufficient strength and damage tolerance are essential to deal with complex stress conditions *in vivo*, including suturing and tearing. Specifically, it exhibits much better mechanical properties, compared with commercially used Bio-Gide membrane (Fig. S8). It is worth mentioning that, compared with the previously reported GBR membranes (such as polycaprolactone (PCL)-based and gelatin-based materials), the tensile strength of the SA-BC membrane in wet environments shows an obvious advantage (Fig. S9) [13,51–54]. Second, its good mechanical stability under wet conditions is critical for bone regeneration, since the regeneration process is an extremely time-consuming task [55–57]. Third, it exhibits excellent flexibility, providing operability for bone regeneration within complex-shaped defects.

**Figure 4.**
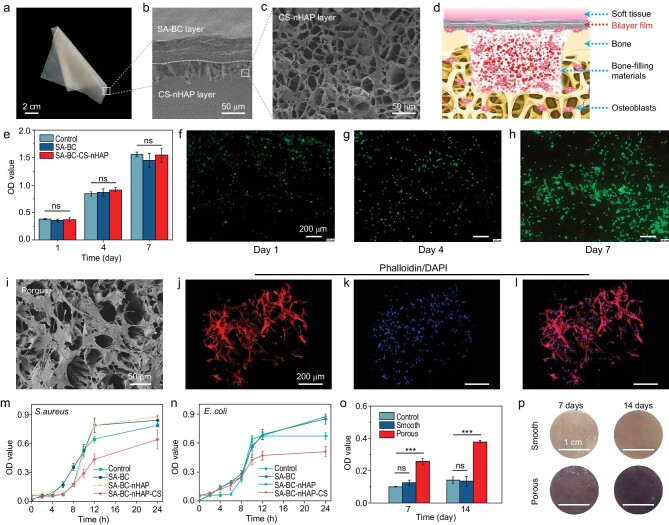
Design and biological evaluation of the bilayer membrane. (a) Photograph of the fabricated bilayer membrane with good flexibility. (b and c) SEM images of the fabricated membrane, presenting a typical closely connected bilayer structure (cross-section view (b)) with a porous surface (exterior view (c)). (d) Schematic illustration of the GBR application of the bilayer membrane. (e) CCK-8 assays of different membranes. (f–h) Live/dead staining images of RBMSCs on the porous surface of the bilayer membrane (green staining indicates viable cells, while red staining indicates dead cells), indicating the bilayer membrane possesses excellent cytocompatibility. (i) SEM image of RBMSCs adhering to the porous surface for 4 days. (j–l) Phalloidin/DAPI staining images of RBMSCs cultured on the porous surface for 4 days (cytoskeleton staining (j), nuclear staining (k) and merged image (l), respectively), showing the pore is beneficial to cell adhesion. (m and n) OD values of *S.aureus* (m) and *E.coli* (n) co-incubated with different membranes for 24 hours, demonstrating the bilayer membrane possesses bacteriostasis. (o) ALP-activity of RBMSCs cultured on the different surfaces of the bilayer membrane for 7 days and 14 days (ns: no significance; ***: *p* < 0.001). (p) Photographs of ALP staining of RBMSCs cultured on the different surfaces of the bilayer membrane for 7 days and 14 days. Both Fig. 4o and p show that the porous layer endows the bilayer membrane with the ability to promote osteogenic differentiation.

To further evaluate the GBR potential of the SA-BC membrane, inspired by the bilayer design of the Bio-Gide membrane and previous work [13], the SA-BC membrane is decorated with a CS-nHAP (hydroxyapatite nanoparticle, Fig. S10) porous microlayer (CS : nHAP = 1 : 1) via blade-coating and ice-templating techniques (Fig. 4a–d and Fig. S11). Surface observation shows that nHAPs are stably embedded in the CS matrix (Fig. S12). The CS-nHAP porous microlayer and SA-BC basal microlayer are tightly interconnected due to the electrostatic attraction between CS (NH_2_) and SA (COO) [58]. The porous microlayer can be tunable with specific thickness (Fig. S13), providing a possibility for customized design. For the designed bilayer membrane, the strong and tough SA-BC microlayer can provide fundamental mechanical support to prevent membrane perforation and rupture, it can also maintain the space for bone repair and serve as a robust barrier (towards soft tissue) to avoid adhesion and invasion of non-osteoblasts. The porous microlayer (towards bone defect) can promote the adhesion and proliferation of osteoblasts to induce osteogenesis.

### Biological evaluation of bilayer membrane

To evaluate the biocompatibility of the bilayer membrane, Cell Counting Kit-8 (CCK-8) assay and live/dead staining of rat bone-marrow stem cells (RBMSCs) on the porous surface are carried out (Fig. 4e–h). The CCK-8 results show that with prolonged co-culture time, the number of cells increased gradually in all groups (Fig. 4e). Moreover, there are no statistical differences in optical density (OD) value among experimental groups (SA-BC-CS-nHAP, SA-BC) and negative control group (without any membrane) at each observation time. These results indicate that the SA-BC membrane and the bilayer membrane possess excellent cytocompatibility. As shown in Fig. 4f–h, the results of live/dead staining show that cell numbers increased with incubation time, also proving the cytocompatibility of the bilayer membrane. Subcutaneous implantation is performed to evaluate *in vivo* histocompatibility. As shown in Fig. S14a, the membrane is inserted between the skin and the muscle on the back of a rat, and the sample including the membrane is taken for hematoxylin and eosin (H&E) staining at 2 and 4 weeks after the operation. Figure S14b and c shows that there is no obvious inflammatory reaction around the membrane. Moreover, the density of inflammatory cells near the membrane decreases from the second week to the fourth week, revealing the favorable biocompatibility of the membrane.

Different morphological surfaces impart the membrane's different cell adhesion abilities. As mentioned above, it is expected that the porous layer is beneficial to osteoblast adhesion while the smooth surface can perform a barrier function and reduce fibroblast attachment. Phalloidin/4′,6-diamidino-2-phenylindole (DAPI) staining of RBMSCs on the porous surface and NIH 3T3 cells on the smooth surface are conducted, followed by SEM observation, to verify cell adhesion. The SEM image demonstrates that plenty of RBMSCs extend filopodia or lamellipodia attaching to the porous layer of the membrane (Fig. 4i). The same phenomenon also can be seen from the images of the Phalloidin/DAPI staining (Fig. 4j–l). By contrast, only isolated sparse NIH 3T3 cells are found on the smooth surface (Fig. S15). Therefore, the bilayer membrane can assist osteoblast attachment due to the porous layer and its high surface-to-volume ratio. At the same time, the smooth surface reduces the interference of fibroblasts. The simple bilayer structure design and derived effects are good for bone repair.

Though significant progress has been achieved in the GBR field, bacterial infection is still a common complication after GBR surgery, which is the major reason for poor bone healing [53]. In this work, CS in the porous layer is expected to endow the membrane with bacteriostasis. Here, we utilize SA-BC membrane, SA-BC-nHAP membrane and SA-BC-CS-nHAP membrane to co-culture with *Staphylococcus aureus* (*S. aureus*) and *Escherichia coli* (*E. coli*), the main representative strains of Gram-positive bacteria and Gram-negative bacteria, respectively, for 24 hours. The bacteriostatic efficacy of the membranes is examined by measuring the OD value at different intervals. As shown in Fig. 4m and n, the OD values of the SA-BC group slightly increased compared with the control group after co-incubating with these bacteria for 24 hours, which demonstrates that SA and BC have no obvious effect on the bacteriostatic activity of the membrane. When nHAP is introduced, there is little change in OD values compared with the SA-BC group, whereas the incorporation of CS shows improved bacteriostatic ability of the membrane. The specific mechanism of the antibacterial effect of CS may be related to the electrostatic interaction between the positive charge of CS and the negative charge on the surface of bacterial cell membranes, which alters the permeability of the cell membrane, resulting in the leakage of proteins and other cellular contents, thus restricting bacteria growth [59,60].

In our experiment, nHAP is incorporated into a porous layer and is expected to promote osteogenic differentiation. Thus, for the sake of evaluating this ability, osteogenesis marker alkaline phosphatase (ALP) [61,62] is selected to investigate the differentiation of RBMSCs on the porous and smooth surfaces of the membrane. Experimentally, RBMSCs are seeded on the porous surface (recorded as P group) and smooth surface (S group) of the membrane, and ALP-activity and ALP-staining are systematically conducted. According to the results of quantitative ALP-activity (Fig. 4o), the OD value of P group is clearly higher (more than 2 times) than that of S group on days 7 and 14. Moreover, from day 7 to 14, the OD value of P group clearly increases while there is little change in S group. These results imply the P group shows higher ALP-activity. Visually, darker ALP-staining is observed in P group and the ALP-staining deepens with the culture time from the day 7 to day 14 but there is no obvious change in S group (Fig. 4p), which is consistent with the results of ALP-activity. All of the above suggests the enhancement of osteogenic differentiation of the porous layer, which is mainly ascribed to the Ca^2+^ released from the incorporated nHAP [11,63].

### Bilayer membrane for GBR *in vivo*

**Figure 5.**
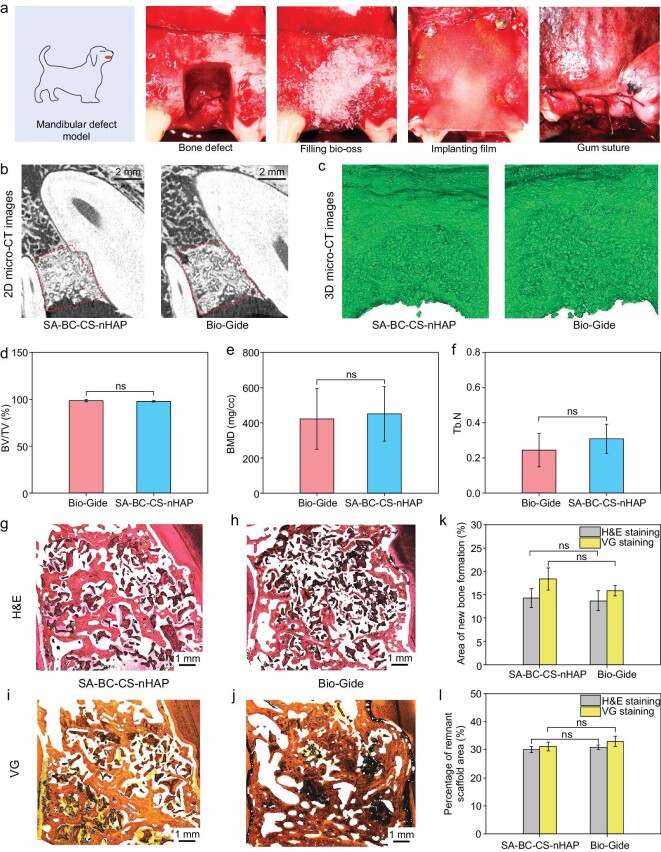
Characterization of bone regeneration *in vivo* of the bilayer membrane. (a) Mandibular defect bone repair experiment. (b and c) 2D micro-CT images and 3D micro-CT images of the new bone formed in the bone defect areas which were treated with the bilayer membrane and Bio-Gide membrane. Qualitatively, there was no significant difference in the amounts of new bone formation between the two groups. (d–f) Bone analysis from micro-CT: new bone volume fraction (d), bone mineralization density (e) and trabecular number (f) (ns: no significance), quantitatively verifying similar osteogenic ability between these groups. (g–j) Histological analysis of new bone in bone defect areas: (g and h) H&E staining images, (i and j) VG staining images. (k and l) Area of new bone formation (k) and percentage of remnant scaffold area (l) calculated from H&E staining and VG staining (ns: no significance).

To further detect the *in vivo* osteogenic potential of the bilayer membrane, a dog mandibular defect model is created. Bio-Gide membrane, the most commonly used GBR membrane in the clinic, is applied as a control. As shown in Fig. 5a, osseous defects (with a length of 15 mm (apical-coronal), a width of 10 mm (mesio-distal) and a thickness of the full layer of the mandible) are prepared and then filled with Bio-Oss (a commonly used bone filling material in the clinic). After that, the bone defects are covered with a SA-BC-CS-nHAP membrane and a Bio-Gide membrane, respectively. Finally, the mucosa is sutured to cover both the defects and the membranes. During these procedures, the SA-BC-CS-nHAP membrane is easy to handle and fits closely with the surrounding tissue which is attributed to its excellent flexibility under wet conditions, while the Bio-Gide membrane is difficult to operate since it became curly when in contact with blood.

After six months, the dogs are sacrificed and specimens are collected for analysis. Typical two-dimensional (2D) micro-computed tomography (micro-CT) images and three-dimensional (3D) images qualitatively manifest that there is no distinct difference in the amount of newly-formed bone observed in the bone defects between SA-BC-CS-nHAP group and Bio-Gide group (Fig. 5b and c). Several indicators, including new bone volume fraction (bone volume/total tissue volume, BV/TV), the bone mineralization density (BMD) and the bone trabeculae number (Tb.N), can also suggest that both the SA-BC-CS-nHAP membrane and the Bio-Gide membrane demonstrate similar ability to quantatively form new bone tissue (Fig. 5d–f). Furthermore, H&E staining and Van Gieson's (VG) staining are performed to visualize the regenerated bone. Judging from the staining results (Fig. 5g–j), the bone formation in the defect treated with the SA-BC-CS-nHAP membrane is similar to or slightly more than that treated with the Bio-Gide membrane. Based on the areas of new bone formation and remnant scaffold calculated from H&E staining and VG staining (Fig. 5k and l), it appears that both types of membrane are equally effective in promoting bone formation. It seems that the histologic results and the BV/TV, BMD, and Tb.N obtained from micro-CT analysis are consistent with each other. All of the above show that the bilayer-designed SA-BC-CS-nHAP membrane exhibits a great potential for GBR.

## CONCLUSION

One mechanical reinforcement strategy based on the HCH dual-scale network is proposed to fabricate strong, tough and damage-tolerant nanocomposites under wet conditions. As one demonstration, the HCH dual-scale network SA@Ca@H_2_O-BC nanocomposite membrane is designed and fabricated. Multiple mechanical studies reveal that the membrane exhibits excellent wet mechanical properties, which is superior to single-scale network SA@Ca@H_2_O membrane and BC@Ca@H_2_O membrane. The SA@Ca@H_2_O molecule-scale network confined in the BC nanofiber-network makes the nanocomposite membrane flexible, while the BC nanofiber-network bears stress for mechanical robustness. The roles of SA@Ca@H_2_O molecule-scale network and BC nanofiber-network are reminiscent of those of small elastic fiber and large collagen fiber found in the dermis of skin [38], reflecting the synergetic effect endowed by the HCH dual-scale network. Based on the mechanically robust nanocomposite membrane, we design a bilayer structure used for GBR with the addition of a CS-nHAP porous microlayer. The dog mandibular defect repair experiment reveals that the SA-BC-CS-nHAP nanocomposite membrane with the integration of mechanical properties and biological functions exhibits great application potential. GBR membrane as a primary demonstration shows that the mechanical reinforcement strategy based on the HCH dual-scale network would provide inspiration to design advanced biomaterials.

## METHODS

### Fabrication of SA@Ca@H_2_O-BC, SA@Ca@H_2_O and BC@Ca@H_2_O membranes

The mixed solution of SA and BC was prepared by mixing 1 wt.% SA (prepared by dissolving 1 g SA powder into 99 ml DIW and stirring until completely dissolved) and 0.5 wt.% BC at a weight ratio of 10 : 4. Then, the solution was cast on the glass slice and the solvent was evaporated naturally to allow the formation of a dense composite membrane. Furthermore, the membrane was soaked into CaCl_2_ solution (1 wt.%) followed by rinsing and drying. Last, the membrane was soaked into DIW for hydration and preservation. For the fabrication of SA@Ca@H_2_O membrane and BC@Ca@H_2_O membrane, the procedures of evaporation, crosslink, and hydration are equally required.

### Mechanical tests

The tensile and suture pullout force tests of the BC-SA membrane were performed on an Instron 5565 A system equipped with a 500 N load cell and 5 mm fixture spacing for tensile tests and a 10 N load cell for suture tests. All membrane samples for the tensile test were cut into rectangles with dimensions of 50 mm × 3 mm and immersed into DIW, PBS and SBF solution, respectively, for 24 h before testing. The tests were performed at a speed of 0.1 mm/sec. During the measurement, a humidifier was used to maintain the humidity of the surrounding environment. At the same time, by carefully dripping water on the samples constantly using a micro-dropper, the membranes were always in a state of liquid infiltration. Thus, the membranes were completely wet. One side of the samples (20 mm length, 10 mm width and 130–165 μm thickness) for testing the suture pullout force was fixed with the clamp of the machine and the other side was fixed to another clamp using a 4–0 (Ethicon) nylon suture which was sewn 2 mm from the edge of BC-SA membrane and the tensile speed was 10 mm/min. For the *in-situ* tensile test, all samples (20 mm length, 10 mm width and 140 ∼150 μm thickness) were prefabricated with cracks of approximately 2 mm in length in the middle, then tested under the *in situ* tensile apparatus (Dual Leadscrew Tensile tester, MT10677) at a speed of 0.5 mm/sec.

## Supplementary Material

nwad333_Supplemental_Files
