# Supplementary material for: Bioinspired polysaccharide-based nanocomposite membranes with robust wet mechanical properties for guided bone regeneration
Source: Natl Sci Rev. 2024 Jan 2;11(3):nwad333. doi: 10.1093/nsr/nwad333 (PMC10852990; doi:10.1093/nsr/nwad333)
Supplement: nwad333_Supplemental_Files [file nwad333_supplemental_files.zip › Supplementary data.pdf]

Supplementary Information

Bioinspired polysaccharide-based nanocomposite membranes with robust wet mechanical properties for guided bone regeneration

Jian-Hong Xiao[1,2,]†, Zhen-Bang Zhang[1,]†, JiaHao Li[3,]†, Si-Ming Chen[1,]*, Huai-Ling Gao[1,3], YinXiu Liao[4], Lu Chen[4], ZiShuo Wang[4], YiFan Lu[2], YuanZhen Hou[3], HengAn Wu[3], DuoHong Zou[2,4,]* and Shu-Hong Yu[1,5,]*

[1]Department of Chemistry, New Cornerstone Science Laboratory, Institute of Biomimetic Materials & Chemistry, Anhui Engineering Laboratory of Biomimetic Materials, Division of Nanomaterials & Chemistry, Hefei National Research Center for Physical Sciences at the Microscale, University of Science and Technology of China, Hefei 230026, China;

[2]Department of Dental Implant Center, Stomatologic Hospital and College, Key Laboratory of Oral Diseases Research of Anhui Province, Anhui Medical University, Hefei 230032, China;

[3]CAS Key Laboratory of Mechanical Behavior and Design of Materials, Department of Modern Mechanics, CAS Center for Excellence in Complex System Mechanics, University of Science and Technology of China, Hefei 230027, China;

[4]Department of Oral Surgery, College of Stomatology, National Clinical Research Center for Oral Diseases, Shanghai Key Laboratory of Stomatology, Shanghai Research Institute of Stomatology, Shanghai Ninth People's Hospital Affiliated to Shanghai Jiao Tong University School of Medicine, Shanghai 200001, China;

[5]Institute of Innovative Materials (I2M), Department of Chemistry, Department of Materials Science and Engineering, Southern University of Science and Technology, Shenzhen 518055, China

***Corresponding authors**. E-mails: smchentj@ustc.edu.cn; zouduohongyy@126.com; shyu@ustc.edu.cn

†Equally contributed to this work.

Materials

nHAPs were purchased from Aladdin, BC aqueous dispersion (0.5 wt.% aqueous dispersion) was purchased from Guilin Qihong technology. CaSi nanofibers were synthesized based on previous work [1]. Modified-Simulated Body Fluid (PH1820) was purchased from PHYGENE. Deionized water (DIW) was used. All the other reagents were purchased from Sinopharm Chemical Reagent.

Fabrication of SA@Ca@H_2O-CaSi membrane

SA solution (1 wt.%) and CaSi dispersion (10 mg/mL) were used and their weight ratio was set as 4:1. Other procedures were similar to those for fabricating BC-based membranes.

Fabrication of SA-BC-CS-nHAP membrane

The mixed dispersion of 2 wt.% CS solution (prepared by dissolving 2g CS powder into 2% v/v acetic acid solution) and 2 wt.% nHAP dispersion (2 g nHAP was evenly dispersed in an aqueous solution by ultrasonication) (weight ratio 1:1) was prepared. Then, it was blade-coated on the surface of the as-obtained SA-BC membrane, and a porous layer was fabricated via an ice-templating procedure. Furthermore, the membrane was sequentially soaked into 0.3 M cold NaOH-ethanol solution (~20 ^{o}C) and $CaCl_2$ solution (1 wt.%), after which the integral functionalized wet membrane was obtained.

Sample characterizations

Scanning electron microscope (SEM) images were obtained by Zeiss Supra 40 at an accelerating voltage of 5 kV. Transmission electron microscope (TEM) images were obtained by Hitachi HT7700 at an accelerating voltage of 200 kV. Energy disperse spectroscopy (EDS)-mapping was used to investigate the element distribution of the bilayer membrane. X-ray diffraction (XRD) pattern was obtained by Philips X'Pert Pro Super X-ray diffractometer with Cu Ka radiation and a scanning speed of 0.2 °min^{-1}. Fourier Transform Infrared Spectrometer (FTIR) spectra were obtained by Nicolet 8700.

Simulations

All the simulations were performed with a Large-scale Atomic/Molecular Massively Parallel Simulator (LAMMPS) [2] and three models were built to simulate the process of H_2O penetration. A

230Å*230Å*43Å box with periodic boundary condition was created and four cellulose fibrils built with Cellulose-Builder [3] were placed along the z direction in the center and fixed in X&Y direction for all three models to prevent unraveling. For the first model (Fig. 2b), 5000 H_2O molecules were placed in the box outside of a 110Å*140Å rectangle extended along Z direction, whose center overlaps the center of the box. For the second model (Fig. 2a), a SA chain containing 10 units was built with CHARMM-GUI [4] first and 124 SA chains with 620 calcium cations were placed in the interspace of CFs randomly. Then thermal annealing of 160ps at 500 K and 1 atm with an isothermal-isobaric (NPT) ensemble was performed to get the SA@Ca-CFs structure. Finally, 5000 H_2O molecules were added into the box the same as the first model. For the third model (Fig. 2c), calcium cations were not put and other settings were the same as the second.

After three models were built, they were minimized with the conjugate gradient (cg) method and then relaxed in an isothermal-isobaric (NPT) ensemble with a temperature of 300 K and pressure of 1 atm. During the relaxation, the number of H_2O molecules in a 95Å*117Å rectangle extended along Z direction was counted every 1 fs to characterize the H_2O molecule dynamic penetration velocity of H_2O molecules. The relaxation was performed for 4 ns with 1 fs timestep and OVITO [5] was used to visualize the process of H_2O molecule dynamic penetration. Eventually, a number of H_2O molecules in different radii of circles extended along Z direction was counted to characterize the distribution of H_2O at the last frame.

Biocompatibility assays

The biocompatibility of the GBR membrane was measured by the Cell Counting Kit-8 (CCK-8, Dojindo, Japan) assay and live/dead staining of rat bone marrow-derived stem cells (RBMSCs) cultured on the porous surface of the membrane. RBMSCs were isolated from the femurs and tibias of 3/4-week-old male Sprague-Dawley (SD) rats. Only cells of passages 3 to 5 were used in this study. NIH 3T3 cells were used as received. Different membranes were punched into disk shapes with 6 mm diameter and then sterilized using a high-pressure steam sterilizer (HVE-50, Hirayama). For the CCK-8 assay,

RBMSCs were first seeded on the 96-well plates, 5000 cells per well, and incubated in Alpha-MEM supplemented with 10% FBS and 1% penicillin/streptomycin in a humidified atmosphere of 5% CO_2 at 37 ^{o}C. After 24 h, when cells were attached to the bottom of the dish, all pieces were placed into wells to coculture with cells for 1 d, 4 d and 7 d. Then the specimens were removed and a 110 μL mixture of the CCK-8 reagent mixed with MEM-Alpha at 1:10 was added to each well and cultivated for another 2 h. Finally, cell viability was measured by the optical density (OD) value at a wavelength of 450 nm using a microplate reader (n=5, and n indicates the number of each group in the 96-well plate). Cell viability on the porous surface was further evaluated by live/dead staining. Specimens were first placed into the 96-well plate, RBMSCs cells were then seeded on the porous surface at a density of 3×10^4 cells. After incubation for 1 d, 4 d and 7 d, live/dead staining was performed using a calcein-AM staining kit (Shanghai Yisheng Biotechnology Co., Ltd., China) and observed by fluorescence microscopy (DM2000, Leica, Germany).

Adhesion experiment

To observe the cell adhesion on different surfaces, RBMSCs and NIH 3T3 cells at a density of 1×10^5 were seeded on the porous and smooth surfaces of the membrane covered on the 24-well plates for 4 d. Afterward, the membranes with cells were washed three times with PBS, then fixed by 4% paraformaldehyde for 40 min, and washed 3~5 times, 3~5 minutes each time with PBS. Thereafter, the F-actin and nuclei were stained with Phalloidin and DAPI respectively. Cell adhesion on the different surfaces was detected by confocal laser scanning microscopy (CLSM, TCS SP8, Leica, Germany).

SEM sample preparation

After coculturing for 4 d, the samples with cells were washed, fixed and dehydrated in ethanol with different concentrations (50%, 60%, 70%, 75%, 80%, 85%, 90%, 95% and 100%, 10 min for each step). Then, the samples were washed three times with tert-butyl alcohol for the replacement of ethanol. Finally, the samples were completely dried using a vacuum freeze-dryer before SEM observation.

Bacteriostasis assessment

Gram-negative *E. coli* (ATCC 25922) and Gram-positive *S. aureus* (ATCC 25923) were selected to assess the bacteriostasis of the membrane and the OD values were measured to investigate this property. Different samples were punched (6 mm diameter), sterilized, and individually placed into 96-well plates and 100 μL bacterial suspensions diluted to 10^5 CFU/mL were added to each well and incubated for 24 h. Finally, the samples were removed and the OD values at a wavelength of 600 nm were measured at predetermined times (0, 2, 4, 6, 8, 10, 12 and 24 h after cultivation) (n=5).

In vitro **osteogenic evaluation**

ALP activity and ALP-staining were carried out to evaluate the osteogenic properties of the prepared membrane. For ALP activity and ALP-staining, RBMSCs at a density of 4×10^4 cells were seeded on the smooth layer and porous layer respectively (n=4) in 24-well plates. After incubation for 7 days and 14 days, ALP activity and ALP-staining were respectively conducted by the guidelines of the ALP detection kit (Beyotime, China) and ALP staining kit (Beyotime, China).

In vivo **biocompatibility experiment**

A subcutaneous implantation experiment in rats was carried out to assess the *in vivo* biocompatibility of the SA-BC-CS-nHAP membrane. The procedure was performed under the approval of the Ethics Committee of Shanghai Jiao Tong University (SH9H-2021-A355-SB) Before the surgery, the samples (size=1×1 cm^2) were sterilized with a high-pressure steam sterilizer and then implanted onto the dorsum of male SD rats weighing ~220 g (the samples are located between the skin and the muscle). After the surgery for 2 w and 4 w, skin biopsies including the membranes and peripheral tissues were obtained for further histological examination. The obtained biopsies were fixed, embedded in paraffin, cut into sections (~4 μm thickness) and then stained with hematoxylin-eosin (H&E) for evaluation.

In vivo **bone formation study**

This study was approved by the Ethics Committee of Shanghai Jiao Tong University (SH9H-2021-A355-SB). All methods were carried out and complied with the Guiding Principles for the Care and Use of Laboratory Animals of Shanghai Jiao Tong University. The research was conducted at

the Research Institute for Animal Breeding and Nutrition, Shanghai Jiao Tong University.

Ten healthy male labradors weighing 20~30 kg with full dentition were selected for this study. Under general anesthesia (intramuscular injection of Zoletil-50 (0.11 mL/kg) to induce anesthesia, 8-10 minutes later, an indwelling needle was placed on the animal and a vein was opened. According to the anesthesia state of the animal, 0.5 mL anesthetic stock solution was diluted 10 times to 5 mL statically and was pushed to stabilize the animal), rectangular critical-size osseous defects, measuring (10±1) mm (mesio-distal) and (15±1) mm (apico-coronal) with the thickness of full layer of mandibular, were prepared at the first premolars bilaterally. Bio-oss (bone meal) was filled in the defects, followed by covered with membranes (one side was covered by the designed membrane, while the other side was covered by the commercial Bio-Gide membrane as control). Titanium screws were used to keep the membranes stable. Finally, the mucosal flaps were put down to thoroughly cover the membranes and then sutured with absorbable sutures. The same surgical procedure was performed for all ten Labrador dogs. Six months later, the dogs were euthanized and bone samples were used for further micro-CT analysis and histological analysis. In terms of histological analysis, the specimens were fixed, washed, dehydrated and embedded. After that, the tissue blocks were cut into 200 μm sections (German EXAKT300CP hard tissue microtome). Then the sections were thinned to 20 μm (German EXAKT400S chip grinding machine) and polished. Next, the sections were stained with H&E and van Gieson's (VG) picrofuchsin for observation of new bone formation.

Statistical analysis

SPSS statistics (version 18.0, IBM Corporation) was used for statistical analysis. All quantitative data were depicted as mean ± standard deviation (SD). One-way analysis of variance (ANOVA) was used to perform statistical analysis, with $P < 0.05$ as statistically significant.

Figures

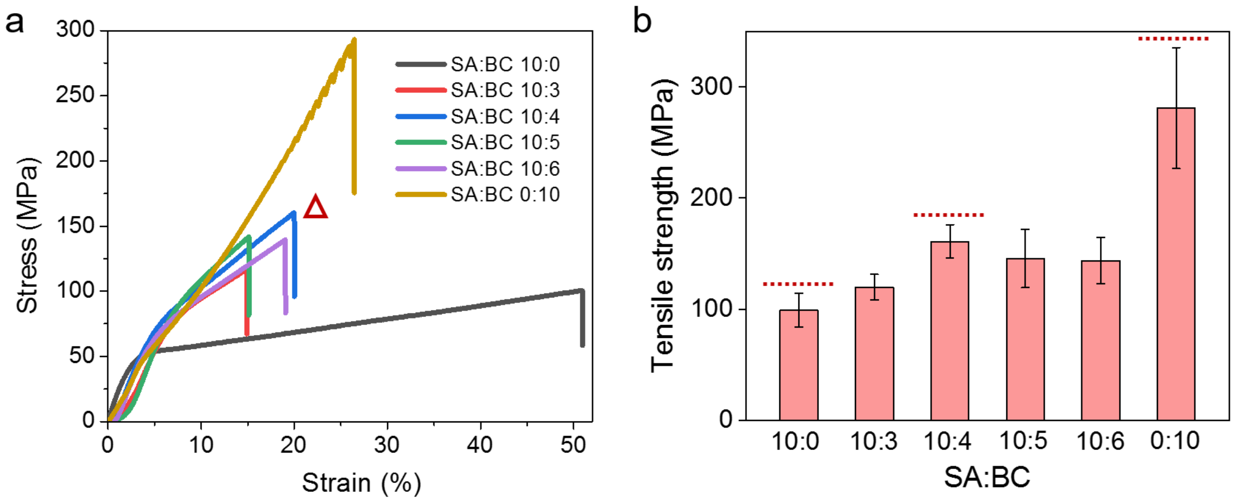

Figure. S1 Mechanical properties of SA-BC membranes in dry state. (a) Typical stress-strain curves of SA-BC membranes with different weight ratios. (b) Tensile strength of SA-BC membranes with different weight ratios, showing that the SA-BC membrane (10:4) has relatively high strength.

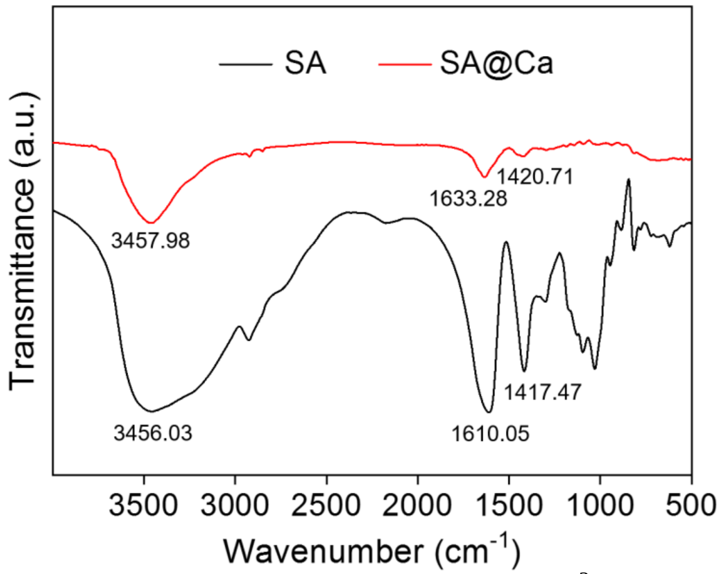

Figure. S2 FTIR spectra of SA and SA@Ca. After introducing Ca^{2+}, the characteristic peaks of COO (asymmetric and symmetric stretching vibrations) in the SA are shifted (from 1610.05 cm^{-1} to 1633.28 cm^{-1}; from 1417.47 cm^{-1} to 1420.71 cm^{-1}), which indicates that Ca^{2+} affects COO. Furthermore, the peak of OH is slightly shifted (from 3456.03 cm^{-1} to 3457.98 cm^{-1}) and deformed, partly implying that Ca^{2+} affects OH.

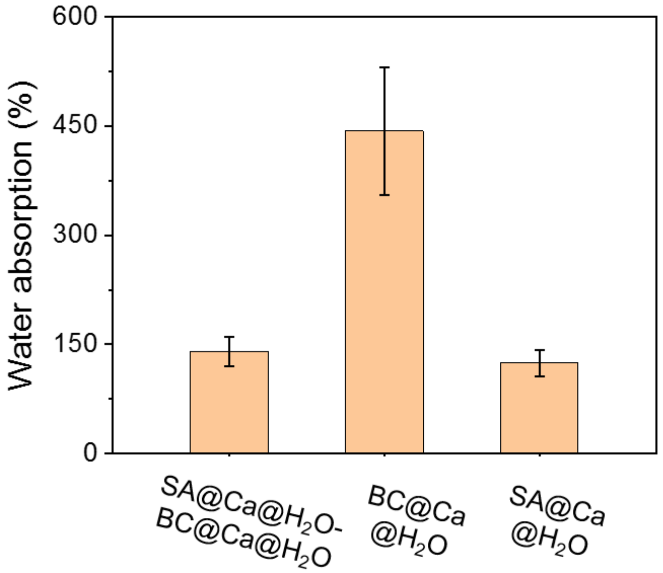

Figure. S3 Water absorption of different membranes.

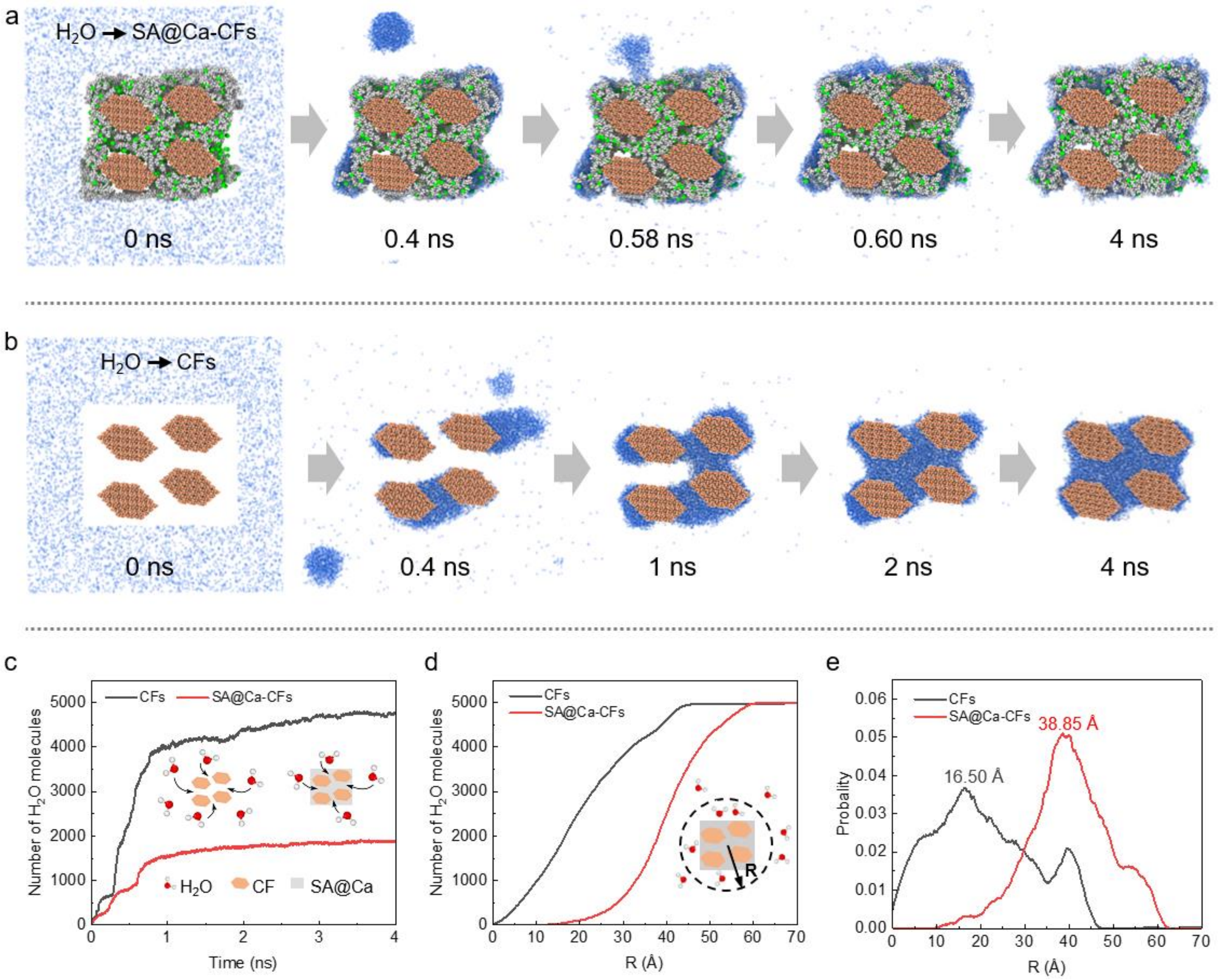

Figure. S4 Water molecules penetration simulations of different systems. The penetration process of SA@Ca-CFs (a) and CFs (b). The green, gray, brown and blue spheres represent Ca^{2+}, SA, CF and H_2O separately. (c) The penetration curves of SA@Ca-CFs and CFs. (d) The distribution of water molecules at 4 ns of SA@Ca-CFs and CFs. (e) The probability distribution of water molecules at 4 ns of SA@Ca-CFs and CFs.

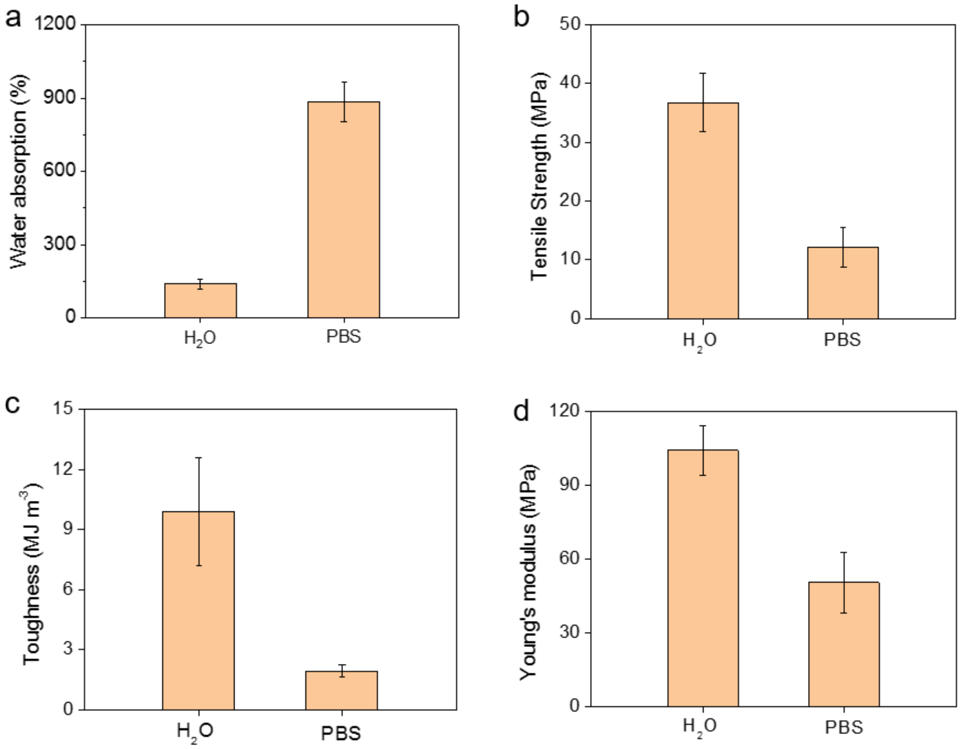

Figure. S5 Comparison of properties of SA-BC membranes in water and PBS. (a) Comparison of the water absorption of SA-BC membranes in water and PBS. (b-d) Tensile strength, toughness and Young's modulus of SA-BC membranes in water and PBS, respectively.

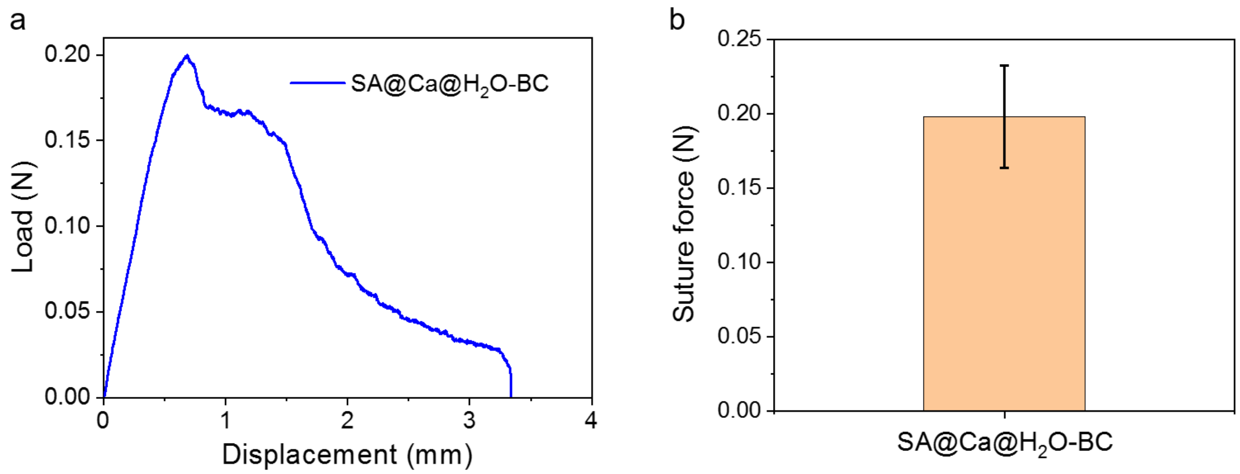

Figure. S6 Wet suture mechanical properties of SA-BC membrane in PBS. Typical load-displacement curve (a) and suture force (b) of the membrane in PBS.

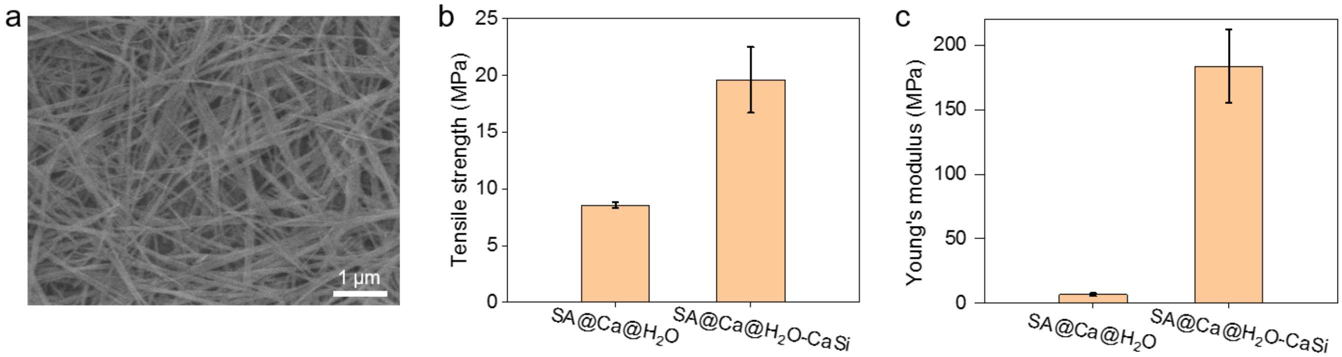

Figure. S7 (a) SEM images of CaSi nanofibers, presenting a similar nanofiber-network structure to BC. (b-c) Comparison of tensile strength and Young's modulus between SA@Ca@H_2O membrane and SA@Ca@H_2O-CaSi membrane, highlighting the superiority of the HCH dual-scale network.

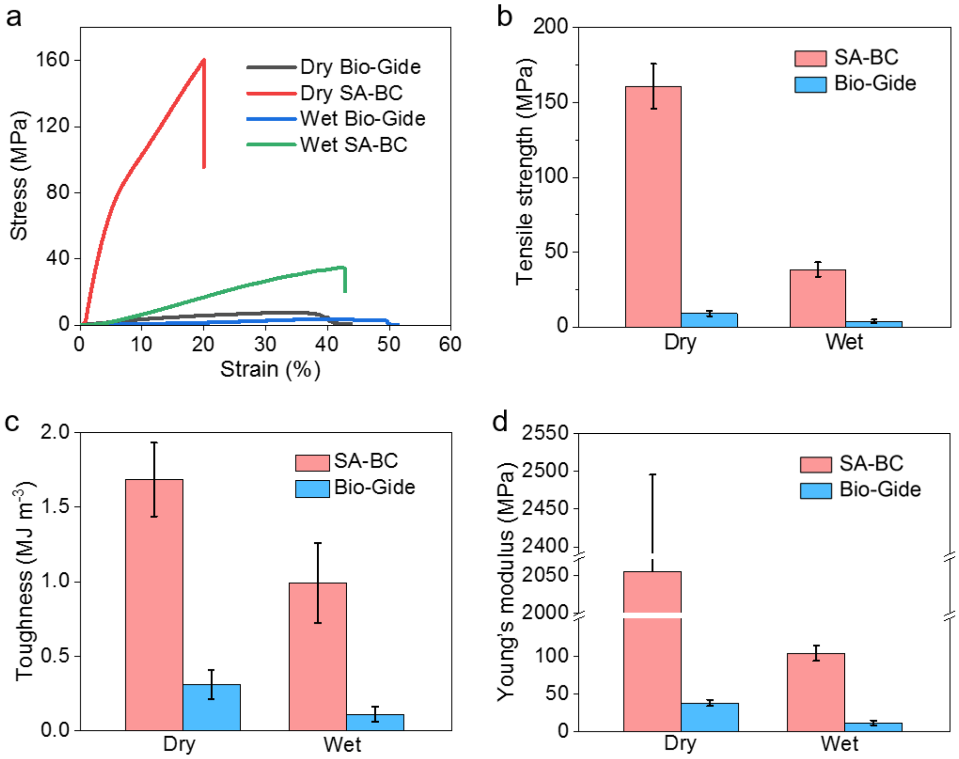

Figure. S8 Mechanical properties of SA-BC membrane and Bio-Gide membrane in both dry and wet states. (a) Typical stress-strain curves of SA-BC membrane and Bio-Gide membrane in dry and wet states. (b-d) Tensile strength, toughness, and Young's modulus of SA-BC membrane and Bio-Gide membrane in dry and wet states, showing that SA-BC membrane exhibits superior mechanical properties to Bio-Gide membrane.

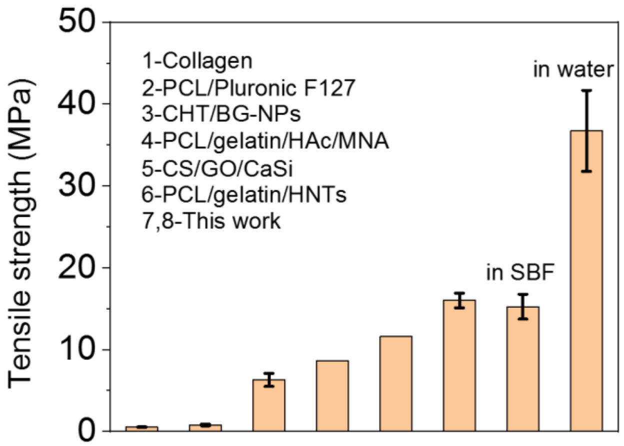

Figure. S9 Comparison of the wet strength of SA@Ca@H_2O-BC membrane (this work) with previously reported membranes. PCL/Pluronic F127 represents polycaprolactone/Pluronic F127, CHT/BG-NPs represents chitosan/bioactive glass nanoparticles, PCL/gelatin/HAc/MNA represents poly(ε-caprolactone)/gelatin/acetic acid/metronidazole, CS/GO/CaSi represents chitosan/graphene oxide/calcium silicate, PCL/gelatin/HNTs represents poly(caprolactone)/gelatin/halloysite nanotubes.

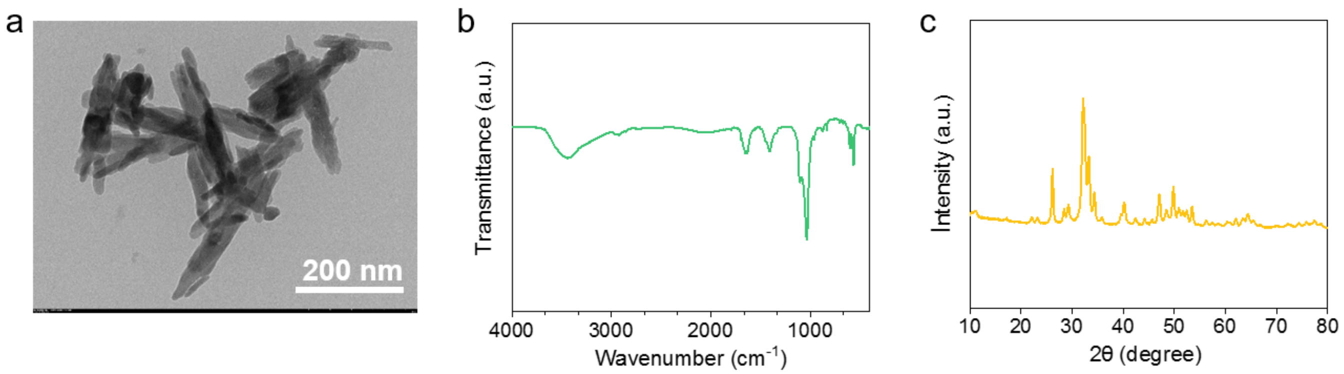

Figure. S10 Characterization of nHAPs. TEM image (a), FTIR spectrum (b) and XRD pattern (c) of nHAPs.

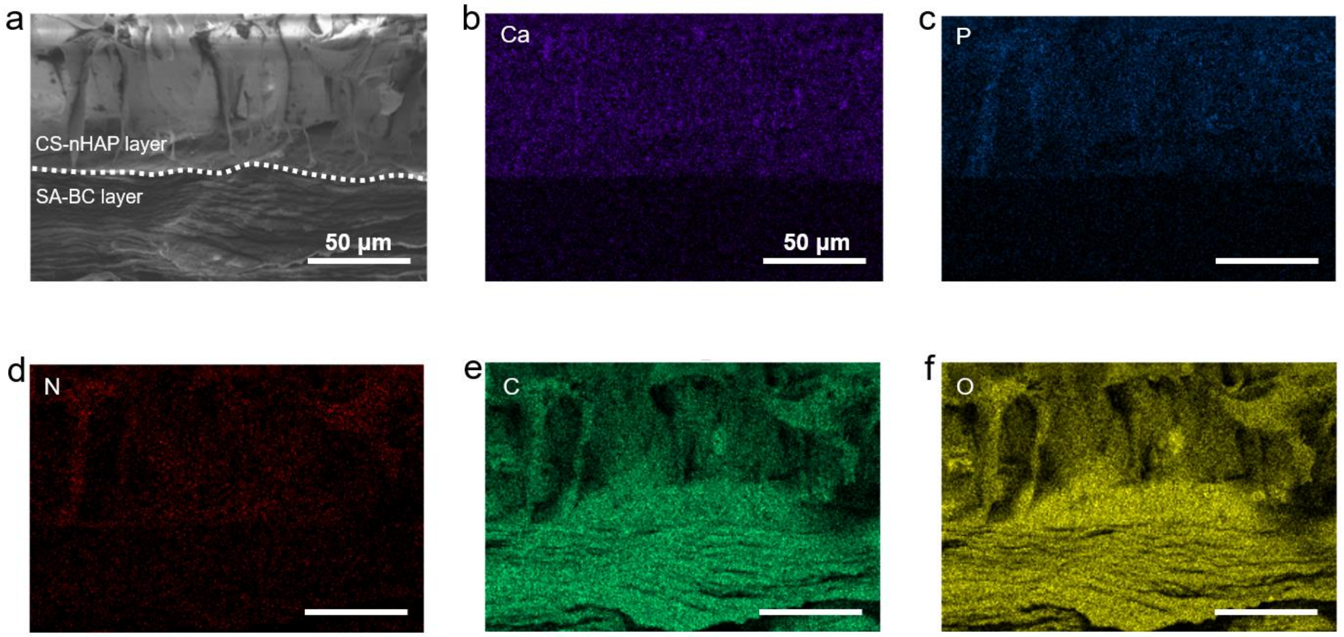

Figure. S11 Element mapping analysis of Ca, P, N, C and O in the side-view of the bilayer membrane.

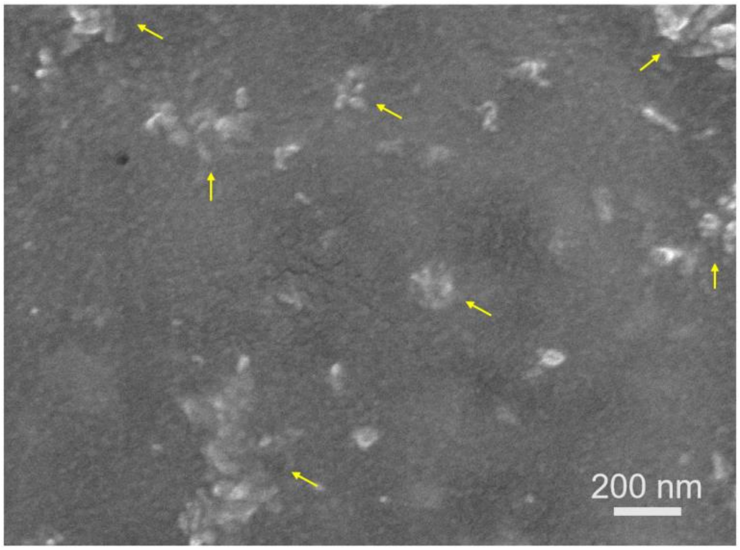

Figure. S12 SEM image of the CS-nHAP layer, showing nHAPs are stably embedded in the CS matrix. Arrows indicate the embedded nHAPs.

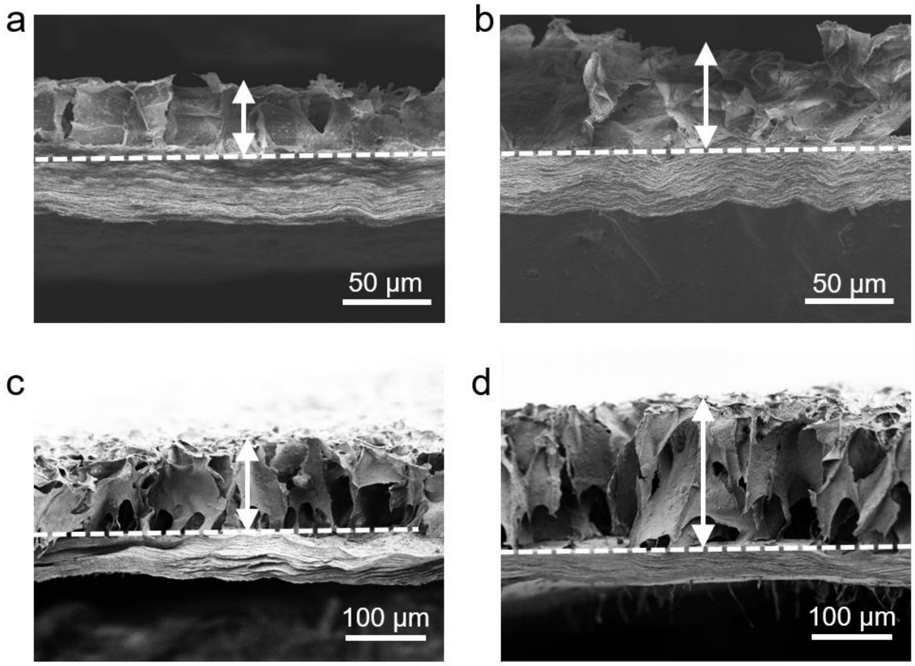

Figure. S13 Bilayer membranes with CS-nHAP porous microlayer of varying thickness, showing flexible design.

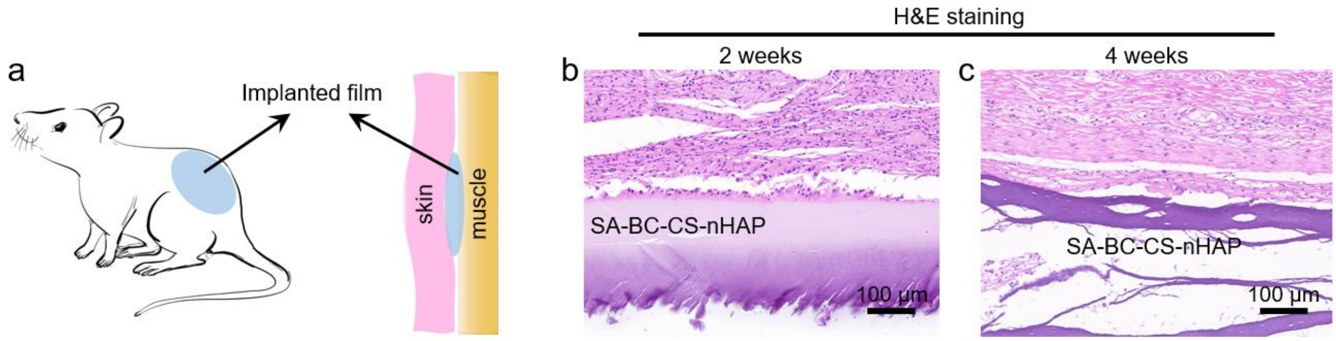

Figure. S14 Characterization of biocompatibility *in vivo* of the bilayer membrane. (a) Schematic diagram of the subcutaneous implantation experiment in the rat. (b-c) H&E staining images at 2 and 4 weeks after the operation, showing the bilayer membrane possesses good histocompatibility.

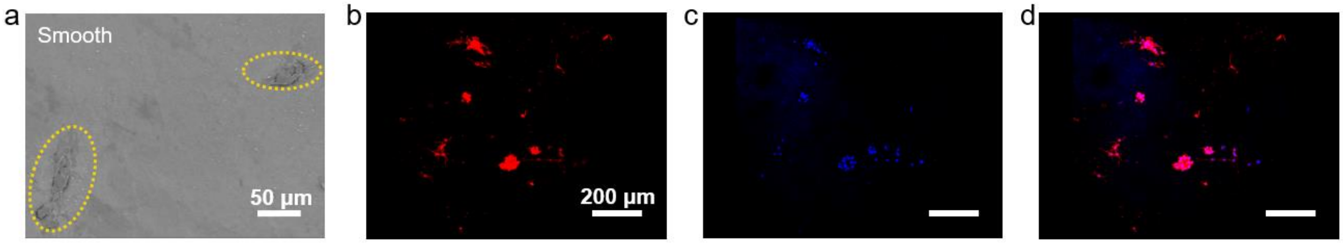

Figure. S15 Cell adhesion on the smooth surface of the bilayer membrane. (a) SEM image of NIH-3T3 cells (yellow circles) adhered to the smooth surface for 4 days. (b-d) Phalloidin/DAPI staining images of NIH-3T3 cells cultured on the smooth surface for 4 days (cytoskeleton staining (b), nuclear staining (c) and merged image (d), respectively), presenting the smooth surface of the bilayer membrane reduces the interference of fibroblasts.

References

1. Lin K, Liu X and Chang J et al. Facile synthesis of hydroxyapatite nanoparticles, nanowires and hollow nanostructured microspheres using similar structured hard-precursors. *Nanoscale* 2011; **3**: 3052-5.
2. Thompson AP, Aktulga HM and Berger R et al. LAMMPS-a flexible simulation tool for particle-based materials modeling at the atomic, meso, and continuum scales. *Comput Phys Commun* 2022; **271**: 108171.
3. Gomes TC and Skaf MS. Cellulose-Builder: A toolkit for building crystalline structures of cellulose. *J Comput Chem* 2012; **33**: 1338-46.
4. Jo S, Kim T and Iyer VG et al. CHARMM-GUI: a web-based graphical user interface for CHARMM. *J Comput Chem* 2008; **29**: 1859-65.
5. Stukowski A. Visualization and analysis of atomistic simulation data with OVITO-the Open Visualization Tool. *Modell Simul Mater Sci Eng* 2009; **18**: 015012.
